# A global spatial analysis reveals where marine aquaculture can benefit nature and people

**DOI:** 10.1371/journal.pone.0222282

**Published:** 2019-10-09

**Authors:** Seth J. Theuerkauf, James A. Morris, Tiffany J. Waters, Lisa C. Wickliffe, Heidi K. Alleway, Robert C. Jones

**Affiliations:** 1 Global Oceans Team, The Nature Conservancy, Arlington, Virginia, United States of America; 2 National Ocean Service, National Oceanic and Atmospheric Administration, Beaufort, North Carolina, United States of America; 3 CSS, Inc. under contract to National Oceanic and Atmospheric Administration, Beaufort, North Carolina, United States of America; 4 Department of Primary Industries and Regions, Government of South Australia, Adelaide, Australia; 5 University of Adelaide, Adelaide, Australia; University of Waikato, NEW ZEALAND

## Abstract

Aquaculture of bivalve shellfish and seaweed represents a global opportunity to simultaneously advance coastal ecosystem recovery and provide substantive benefits to humanity. To identify marine ecoregions with the greatest potential for development of shellfish and seaweed aquaculture to meet this opportunity, we conducted a global spatial analysis using key environmental (e.g., nutrient pollution status), socioeconomic (e.g., governance quality), and human health factors (e.g., wastewater treatment prevalence). We identify a substantial opportunity for strategic sector development, with the highest opportunity marine ecoregions for shellfish aquaculture centered on Oceania, North America, and portions of Asia, and the highest opportunity for seaweed aquaculture distributed throughout Europe, Asia, Oceania, and North and South America. This study provides insights into specific areas where governments, international development organizations, and investors should prioritize new efforts to drive changes in public policy, capacity-building, and business planning to realize the ecosystem and societal benefits of shellfish and seaweed aquaculture.

## Introduction

Globally, coastal ecosystems face numerous complex and interconnected anthropogenic threats, such as nutrient pollution, loss of habitats, and the compounding impacts of climate change [1]. These stressors can challenge or change the way in which ecosystems provide vital services, such as nutrient cycling or maintenance of fisheries, to coastal communities. As the global population grows, the demand for resources from coastal ecosystems, including seafood products, is increasing [2,3]. With growing consumption of seafood, plateauing wild fishery harvests, and apparent limits to land-based agriculture, aquaculture is one of the fastest growing forms of food production on the planet [2,4,5]. While aquaculture’s rapid rise has in many cases coincided with negative localized impacts on surrounding ecosystems [6], evidence increasingly indicates that aquaculture production of certain species groups, such as bivalve shellfish and seaweeds, has the potential to provide valuable ecosystem services that may allow aquaculture to serve as a tool to facilitate coastal ecosystem recovery [7].

When managed within a broader ecosystem framework and strategy, aquaculture has the potential to enhance ecosystems and provide increased benefits to humanity [8], with values potentially returned through a wide range of regulating, provisioning, habitat, and cultural ecosystem services [9]. Aquaculture includes a diversity of activities and objectives—including ‘industrial’-scale food production, specialized operations focused on environmental outcomes or multi-species production for broader benefits, and native species restoration [8,9]. By actively designing aquaculture to deliver ecosystem services, it may be possible to achieve greater positive impact on ecological, economic, and social needs through enhanced habitat restoration, increased employment, and increased food security, respectively. We refer to the intentional use of aquaculture to positively affect these services as ‘restorative aquaculture.’ For example, seaweed aquaculture, through photosynthetic uptake of carbon dioxide, can mitigate local (kilometer-scale) effects of increased ocean acidification by increasing the aragonite saturation state [10]. Many bivalve shellfish and seaweed species assimilate nutrients from surrounding waters [11,12], yielding improvements to water quality and clarity [13,14]. Mussel, clam, and oyster aquaculture can provide fish habitat and enhanced benthic community diversity and production [15,16,17,18]. Bivalve shellfish and seaweed aquaculture can also support a myriad of cultural ecosystem services and societal benefits, such as improved food security, novel forms of employment, and opportunities for improved gender equity [19,20,21]. The direction and magnitude of shellfish and seaweed aquaculture’s benefits to, and impacts on, ecosystem services and society ultimately depend on the coalescence of a wide range of farm-, regional-, and biogeographical-scale environmental and socioeconomic conditions, such as the species farmed, cultivation method, hydrodynamics, nutrient status of the ecosystem, and/or the ability to effectively manage the aquaculture sector [9].

Habitat-forming ecosystem engineers, such as oyster reefs, seagrass beds, and kelp forests, and associated biotic assemblages provide critical ecosystem services, but have suffered extensive losses globally as a result of overharvest, nutrient pollution, and other detrimental human activities [22,23,24]. One of the most dramatic examples is the more than 85% global loss of oyster reefs within the past two centuries [22]. Significant efforts led by governments and nongovernmental organizations are underway to restore coastal habitats such as oyster reefs and kelp forests to recover ecosystem services [22,24]. However, the substantial cost (e.g. US$80,000 to US$1,600,000 per hectare) and varied success (38.0%– 64.8% survival two years post-restoration) has led to growing recognition of the need to identify novel tools and market-driven funding sources to recover ecosystems [25]. Market-based solutions—such as development of commercial shellfish and seaweed aquaculture industries—may be particularly valuable to aid ecosystem recovery where public funds to support coastal restoration efforts are limited or unavailable. This could be an important pathway for low or lower-middle income nations to initiate or grow aquaculture production to meet socioeconomic (e.g., food security, job creation) and environmental objectives (e.g., habitat enhancement).

While commercial shellfish and seaweed aquaculture can help address a multitude of global environmental challenges and social issues, a variety of societal and human health factors also restrict where safe or sustainable shellfish and seaweed aquaculture can be developed. For example, regions lacking wastewater treatment infrastructure or where heavy metal or persistent organic pollutant concentrations are elevated may lead to production of shellfish or seaweed unfit for human consumption [26]. Additionally, where the ability of governments to formulate, implement, and enforce sound policies and regulations is inadequate, it may not be possible to manage shellfish and seaweed aquaculture to ensure sustainability of the sector [27].

In this study, we conducted a global-scale spatial analysis that incorporated a broad suite of global datasets representing key environmental, socioeconomic, and human health considerations to develop a novel, quantitative global index—the ‘Restorative Aquaculture Opportunity Index’ (RAOI)—to aid in identification of marine ecoregions with substantial opportunity to benefit from ecosystem services provided by development of restorative shellfish and seaweed aquaculture (hereafter termed ‘high opportunity marine ecoregions,’ or HOMEs). Specifically, we: (1) convened an expert panel of stakeholders to identify model inputs to the RAOI and weight their relative importance, (2) used a GIS-based modeling approach to integrate spatial data layers representing 16 environmental, socioeconomic, and human health factors to calculate the RAOI under varying scenarios optimized for shellfish and seaweed aquaculture, and (3) assessed sensitivity of the RAOI to the expert weightings and to individual factors. In this study, we identified regions of high opportunity around the world where commercial marine aquaculture may provide environmental and social benefits to drive conservation, public policy, and business planning efforts towards aquaculture systems that enhance ecosystem function, potentially unlocking a pragmatic approach to ecosystem recovery. To illustrate the immediate opportunity available to use restorative aquaculture in this way, we describe the efforts underway to operationalize the results of this study to guide shellfish and seaweed aquaculture development projects around the world.

## Methods

### Identification of applicable factors

An initial literature review was conducted to understand the broad set of environmental, socioeconomic, and human health dimensions that might define where development of shellfish and seaweed aquaculture could mitigate environmental challenges and ensure societal benefits while accounting for constraints to sustainable development. In particular, we utilized resources such as the recent systematic literature review of ecosystem services associated with aquaculture conducted to determine applicable factors for inclusion within the analysis [7]. Concurrently, we conducted a thorough review of global datasets from authoritative sources (e.g., United Nations, World Bank) to determine which of the applicable factors had sufficient data available at the global scale. Where data were unavailable through central sources, new global spatial datasets were compiled from published resources. For example, global seagrass habitat loss data were sourced from a publication’s supplemental materials and global harmful algal bloom occurrences data were sourced from records provided by the Intergovernmental Oceanographic Commission of the United Nations Educational, Scientific and Cultural Organization [23,28]. All data sources, along with a rationale for inclusion and description, are provided within Table 1. To ensure the spatial analysis did not incorporate duplicative factors, we conducted a regression analysis to determine if statistically significant correlations existed between spatial datasets. Where correlations existed amongst spatial datasets representing individual factors (e.g., correlation between the World Bank’s World Governance Indicators ‘regulatory quality’ and ‘political stability’), we incorporated the dataset that was determined to be most authoritative and applicable.

**Table 1:** List of 16 factors represented by spatial data layers used to determine high opportunity marine ecoregions for shellfish and seaweed restorative aquaculture development. A score of 100 denotes the highest opportunity for a given factor for restorative aquaculture, whereas a score of 0 denotes the lowest opportunity.

Factor	Rationale	Description	Suitability Relationship	Data Source
***Environmental***				
Nutrient Pollution	Shellfish and seaweed aquaculture can mitigate effects of nutrient pollution through filtration of phytoplankton, enhanced benthic denitrification, and biomass assimilation of nutrients	Estimated change in coastal discharge of dissolved inorganic nitrogen (DIN) to marine ecoregions between preindustrial and contemporary times	Log-transformed change in DIN loadings standardized on a 0 (decrease or lowest increase) to 100 (highest increase) scale	Hoekstra JM, Molnar JL, Jennings M, Revenga C, Spalding MD, Boucher TM, et al. The atlas of global conservation: Changes, challenges and opportunities to make a difference. Berkeley: University of California Press; 2010. Green PA, Vörösmarty CJ, Meybeck M, Galloway JN, Peterson BJ, Boyer EW. Pre-industrial and contemporary fluxes of nitrogen through rivers: A global assessment based on typology. Biogeochemistry. 2004;68: 71–105.
Habitat Loss (Oyster Reefs)	Shellfish and seaweed aquaculture can provide habitat where oyster reefs have degraded	Remaining extent of oyster reefs relative to historic distribution	100: <1% of historic abundance 66: 2–10% remain 33: 11–50% remain 0: 50+% remain	Beck MW, Brumbaugh RD, Airoldi L, Carranza A, Coen LD, Crawford C, et al. Oyster reefs at risk and recommendations for conservation, restoration, and management. BioScience. 2011;61: 107–116.
Habitat Loss (Kelp Forests)	Shellfish and seaweed aquaculture can provide habitat where kelp forests have degraded	Percent change in annual kelp forest extent over the past 50 years	100: >3% annual loss 66: -1 - -3% loss 33: 0 - +1% expansion 0: >1% expansion	Krumhansl KA, Okamoto DK, Rasswiler A, Novak M, Bolton JJ, Cavanaugh KC, et al. Global patterns of kelp forest change over the past half-century. Proceeding of the National Academy of Sciences. 2016;113: 13785–13790.
Habitat Loss (Seagrass Beds)	Shellfish and seaweed aquaculture can provide habitat where seagrass beds have degraded	Remaining extent of seagrass beds relative to historic distribution	100: >25% loss relative to historic abundance 66: 10–25% loss 33: 10% loss– 10% expansion 0: >10% expansion	Waycott M, Duarte CM, Carruthers TJB, Orth RJ, Dennison WC, Olyarnik S, et al. Accelerating loss of seagrasses across the globe threatens coastal ecosystems. Proceedings of the National Academy of Sciences. 2009;106: 12377–12381.
Ocean Acidification	Seaweed aquaculture can buffer against local effects of ocean acidification	As one of the more soluble forms of calcium carbonate, aragonite saturation state is a common indicator of ocean acidification vulnerability	Aragonite saturation state values linearly transformed and standardized on a 0 (highest) to 100 (lowest) scale	Jiang LQ, Feely RA, Carter BR, Greeley DJ, Gledhill DK, Arzayus KM. Climatological distribution of aragonite saturation state in the global oceans. Global Biogeochemical Cycles. 2015;29: 1656–1673.
Trawl Fishing Pressure	Shellfish and seaweed aquaculture, through habitat provision for juvenile fish and substitutability of aquaculture-produced seafood products, can combat excess fishing pressure	Trawling and dredging fishing pressure by marine ecoregion between 1955–2004, inclusive of fish and shellfish harvest	Log-transformed fish landings (tonnes) standardized on a 0 (lowest bottom trawl landings) to 100 (highest) scale	Watson R, Revenga C, Kura Y. Fishing gear associated with global marine catches I: Database development. Fisheries Research. 2006;79: 97–102.
***Socioeconomic***				
Aquaculture Value (Shellfish)	Emphasis should be placed on promoting growth of shellfish aquaculture where it has recently occurred profitably	Mean total production value by country from 2011–2015	Log-transformed total production value standardized on a 0 (no production) to 100 (highest production) scale	United Nations Food and Agriculture Organization. Global Aquaculture Production Dataset. Rome; 2016.
Aquaculture Value (Seaweeds)	Emphasis should be placed on promoting growth of seaweed aquaculture where it has recently occurred profitably	Mean total production value by country from 2011–2015	Log-transformed total production value standardized on a 0 (no production) to 100 (highest production) scale	United Nations Food and Agriculture Organization. Global Aquaculture Production Dataset. Rome; 2016.
Regulatory Quality	Emphasis should be placed on promoting growth of shellfish and seaweed aquaculture where regulatory quality is sufficient to manage the sector	Relative rank by country for regulatory quality, inclusive of perceptions of the ability of the government to formulate and implement sound policies and regulations	Regulatory quality rank linearly transformed and standardized on a 0 (lowest rank) to 100 (highest) scale.	Kaufmann D, Kraay A, Mastruzzi M. The worldwide governance indicators: Methodology and analytical issues. *World Bank Policy Research Working Paper No*. *5430*. 2010.
Food Security	Emphasis should be placed on promoting growth of shellfish and seaweed aquaculture where it can contribute to enhancing food security	Relative rank by country for food security, inclusive of affordability, availability, and quality	Food security rank linearly transformed and standardized on a 0 (highest rank) to 100 (lowest) scale.	The Economist Intelligence Unit. Global Food Security Index 2017: Measuring food security and the impact of resources risks. 2017.
Logistics Performance	Emphasis should be placed on promoting the growth of shellfish and seaweed aquaculture where sufficient infrastructure and shipment logistics capacity exist to support the sector	Relative rank by country for logistics performance, inclusive of efficiency of customs, quality of trade/transport infrastructure, ease of arranging shipments, and other logistics considerations	Logistics performance rank linearly transformed and standardized on a 0 (lowest rank) to 100 (highest) scale	Arvis J, Ojala L, Wiederer C, Shepherd B, Raj A, Dairabayeva K, et al. Connecting to compete 2018: Trade logistics in the global economy; The logistics performance index and its indicators. Washington: The World Bank; 2018. pp. 82.
***Human Health***				
Harmful Algal Blooms (HABs)	The past occurrence of HABs can be an indicator of potential for future HABs, some of which can result in human illness with consumption of impacted shellfish	Global reports of HABs, based on aggregated reporting from 1900s to present	Log-transformed total count of HAB records by ecoregion standardized on a 0 (most records) to 100 (fewest) scale	IODE-UNESCO. Harmful Algae Event Database. Oostende. 2019.
Wastewater Treatment	Coastal countries lacking wastewater treatment infrastructure may have sewage contamination of coastal waters that could impact products of shellfish and seaweed aquaculture	Level of wastewater treatment (percentage of water treated) per country normalized by connection rate (percentage of population connected to wastewater treatment)	Wastewater treatment level linearly transformed and standardized on a 0 (lowest level) to 100 (highest) scale	Malik OA, Hsu A, Johnson LA, de Sherbinin A. A global indicator of wastewater treatment to inform the Sustainable Development Goals (SDGs). Environmental Science and Policy. 2015;48: 172–185.
Persistent Organic Pollutants (POPs)	Shellfish and seaweed produced through aquaculture can bioaccumulate POPs, posing potential human health risks	Global reports of concentrations of persistent organic pollutants (i.e., DDT, PCB, HCH) as indicated by concentrations on resin pellets	Log-transformed average concentration of POPs standardized on a 0 (highest concentration) to 100 (lowest) scale	Ogata Y, Takada H, Mizukawa K, Hirai H, Iwasa S, Endo S. International pellet watch: Global monitoring of persistent organic pollutants (POPs) in coastal waters. 1. Initial phase data on PCBs, DDTs and HCHs. Marine Pollution Bulletin. 2009;58: 1437–1446.
Mercury	Shellfish and seaweed produced through aquaculture can bioaccumulate mercury, posing potential human health risks	Global mercury deposition to coastal waters (seawater concentration)	Average seawater mercury concentration within an ecoregion standardized on a 0 (highest concentration) to 100 (lowest) scale	United Nations Environment Program, Arctic Monitoring and Assessment Programme (UNEP-AMAP). Global mercury modelling: Update of modelling results in the global mercury assessment 2013. Oslo: 2015. pp. 32.
Microplastics	Shellfish can ingest and accumulate microplastics, yielding potential human health impacts to consumers	Global modeled dataset on microplastic concentrations	Average seawater microplastic concentration within an ecoregion standardized on a 0 (highest concentration) to 100 (lowest) scale.	van Sebille E, Wilcox C, Lebreton L, Maximenko N, Hardesty BD, van Franeker JA, et al. A global inventory of small floating plastic debris. *Environmental Research Letters*. 2015;10: 124006.

### Expert stakeholder input to guide determination of factors and their weightings

We engaged a 13-person stakeholder group to determine factors to include within the RAOI and their associated weightings. The stakeholder group was purposively selected to consist of international expert representatives across the shellfish and seaweed aquaculture sectors, government scientists, private industry, academic and research institutions, non-governmental organizations, and international financial institutions (inclusive of representation from Australia, New Zealand, Norway, and the United States of America). The stakeholder group determined which factors were to be included in the RAOI (Table 1) and independently assigned individual quantitative weights to each based on their perceived importance of a given factor in identifying HOMEs for development of commercial shellfish or seaweed aquaculture (higher weight = more important, all weights sum to 100%). Weightings were assigned separately for shellfish- and seaweed-optimized scenarios of the RAOI. For environmental-, socioeconomic-, and human health-optimized scenarios of the RAOI, we re-scaled the assigned weightings factors within these categories such that they summed to 100% within a given category. The weightings that were ultimately applied to the factors used to compute the RAOI represented the average weighting assigned for a given factor across all individuals within the stakeholder group (Table 2). The integration of stakeholder input on factors, weights and criteria did not require the review of an institutional review board as the information that was collected does not meet the definition of human research as defined by federal regulations. Specifically, the information that was collected was not about the participants nor did it manipulate their environment.

**Table 2:** Factors and associated weights utilized to compute the restorative aquaculture opportunity index (RAOI) in the six analysis scenarios. Weights were applied to each factor, and the assigned weight corresponds to the relative importance of each factor in determining high opportunity marine ecoregions for shellfish and/or seaweed restorative aquaculture development in a given analysis scenario. Note that for habitat loss, the indicated weight was divided across all three habitat types (oyster, kelp, and seagrass). The assigned weight of all factors in each scenario sum to 100%.

Factors	All Factors, Shellfish	All Factors, Seaweed	Environmental, Shellfish	Environmental, Seaweed	Socioeconomic	Human Health
***Environmental***						
Nutrient Pollution	17.44%	14.69%	43.07%	36.28%	-	-
Habitat Loss (Oyster, Kelp, Seagrass)	16.27%	13.70%	40.18%	33.85%	-	-
Ocean Acidification	-	6.38%	-	15.77%	-	-
Trawl Fishing Pressure	6.78%	5.71%	16.74%	14.10%	-	-
***Socioeconomic***						
Aquaculture Value (Shellfish)	10.63%	-	-	-	17.43%	-
Aquaculture Value (Seaweeds)	-	10.63%	-	-	17.43%	-
Regulatory Quality	7.78%	7.78%	-	-	25.53%	-
Food Security	5.68%	5.68%	-	-	18.64%	-
Logistics Performance	6.39%	6.39%	-	-	20.97%	-
***Human Health***						
Harmful Algal Blooms	8.67%	-	-	-	-	29.86%
Wastewater Treatment	7.56%	12.06%	-	-	-	26.04%
Persistent Organic Pollutants	6.66%	10.63%	-	-	-	22.94%
Mercury	3.97%	6.34%	-	-	-	13.68%
Microplastics	2.17%	-	-	-	-	7.48%

### Assembly, processing and standardization of spatial data

For each environmental, socioeconomic, and human health factor for which an authoritative global dataset was identified, we followed a standardized protocol to process each dataset within ArcMap 10.5 for integration within the RAOI [29]. Each global dataset was summarized to the finest common spatial scale—marine ecoregions (MEs) [30]. MEs are the finest-scale, standardized biogeographic classification system available for coastal systems globally and represent 232 distinct coastal areas (inclusive of multiple sub-estuaries) sharing common biogeographic traits, such as isolation, upwelling, nutrient inputs, freshwater influx, temperature regimes, ice regimes, sediments, currents, and bathymetric or coastal complexity. For datasets that contained multiple values within an ME, we calculated the average value for each ME. For datasets available at a country-level, we calculated the proportion of each country’s exclusive economic zone contained within a given ME and assigned a proportional average value to each ME. For some factors, data gaps were present within some MEs—handling of missing data is described in detail within ‘Methods: Caveats’ section.

We subsequently defined a suitability function to describe the relationship between each factor and associated opportunity for development of commercial shellfish and seaweed aquaculture (Table 1). We utilized a linear rescaling approach to convert values for a given spatial data layer onto the common suitability scoring scale (0–100; *sensu* [31]). Linear rescaling was utilized as it requires the fewest assumptions with regards to the form of the function utilized to develop the suitability relationship and simply assigns, for example, the highest possible score (100) to those MEs where an environmental or social challenge that commercial shellfish or seaweed aquaculture could combat was greatest, and the lowest possible score (0) to where these challenges were least. Where data for an individual factor were not normally distributed, log-transformations were required to develop an appropriate suitability function (Table 1). Our underlying assumption through application of these relationships was that HOMEs for development of bivalve shellfish and seaweed aquaculture are those where the enabling conditions for development or environmental challenges that aquaculture can ameliorate are greatest. For example, we defined a suitability relationship for nutrient pollution that assigned MEs with the highest increase in anthropogenic dissolved inorganic nitrogen (DIN) loadings the score corresponding with the highest opportunity (100), and those with the lowest increase in anthropogenic DIN loadings the score corresponding with the lowest opportunity (0). For human health factors, suitability relationships assigned the highest possible score to those MEs where human health risks were minimized, and the lowest possible score to those MEs where risks were greatest.

It is important to note that these suitability functions are intended to represent commercial shellfish and seaweed aquaculture suitability relationships at the scale of MEs (i.e., inclusive of multiple sub-estuaries). When applied at the scale of estuaries for localized spatial planning and siting of specific shellfish and seaweed aquaculture operations, these functions may require modification due to considerations relevant at finer spatial scales (e.g., locations of known hypoxia or anoxia within eutrophic estuaries, locations of known human health risks such as wastewater outfalls).

### Model calculation

**Fig 1:**
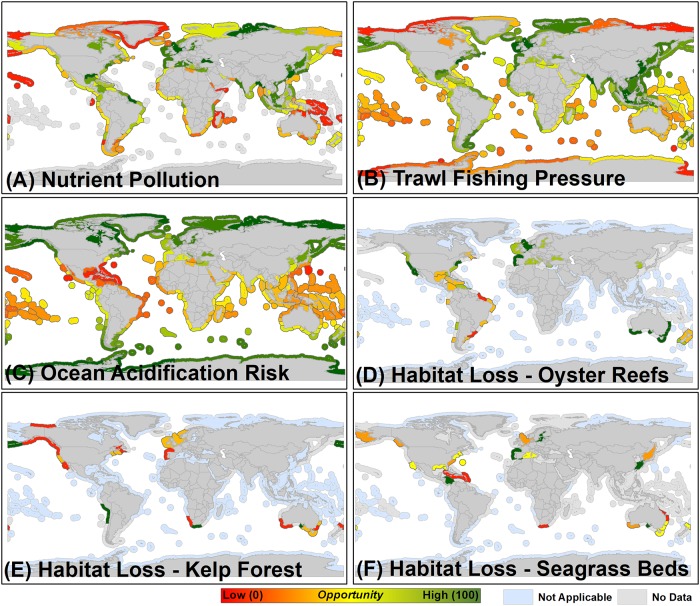
Environmental factors integrated within the restorative aquaculture opportunity index used in determining high opportunity marine ecoregions for shellfish and seaweed restorative aquaculture development, including (A) nutrient pollution, (B) trawl fishing pressure, (C) ocean acidification risk, (D) oyster reef habitat loss, (E) kelp forest habitat loss, and (F) seagrass bed habitat loss. All factors are re-scaled to represent where shellfish and seaweed aquaculture development would provide maximum benefits (see Table 1). For example, marine ecoregions with the greatest oyster reef habitat loss (B) are re-scaled to represent the highest opportunity ecoregions (dark green) where shellfish and seaweed aquaculture could provide habitat enhancement benefits.

Within ArcMap 10.5 [29], the RAOI score for each marine ecoregion (*E*_*j*_) for shellfish and seaweed aquaculture was calculated as:

$$E_{j}=\sum_{x=1}^{n}\left(S_{ij}∙W_{i}\right)$$

where *E*_*j*_ is the cumulative RAOI score of ecoregion *j* calculated as the product of the suitability score *S* of ecoregion *j* for spatial data layer *x* and the weight *W* of layer *i* summed across all *n* layers (e.g., 14 for the shellfish RAOI scenario that incorporated all environmental, socioeconomic, and human health factors; Table 2). On a scale of 0 to 100, ecoregion RAOI scores were ranked from lowest to highest opportunity for development of shellfish and seaweed aquaculture. We computed six unique RAOI scenarios based on the expert panel-selected factors and associated weights (Table 2) to: (1) identify HOMEs based on scenario-specific criteria (e.g., HOMEs based on all environmental, socioeconomic, and human heath factors relevant to shellfish aquaculture), and (2) to better understand drivers of consistency or variation in identified HOMEs. Scenarios evaluated included: (1) all environmental, socioeconomic, and human health factors relevant to shellfish aquaculture, (2) all environmental, socioeconomic, and human health factors relevant to seaweed aquaculture, (3) all environmental factors relevant to seaweed aquaculture, (4) all environmental factors relevant to shellfish aquaculture, (5) all socioeconomic factors, and (6) all human health factors. If a factor was not applicable to a given ME, weightings used to compute the RAOI were adjusted to only account for the inclusion of applicable factors. For example, for the oyster reef, kelp forest, and seagrass bed habitat degradation factors, RAOI scores within a given scenario were calculated for MEs where these habitats have not previously existed (e.g., MEs listed as ‘Not Applicable’ within Fig 1D, 1E and 1F) by proportionally re-scaling the weights assigned to factors applicable to a given ME such that they summed to 100.

### Sensitivity analysis

To determine the sensitivity of the RAOI to assigned weightings, we ran each RAOI scenario with equal weightings for each factor incorporated into each respective scenario. We visually compared output from each expert-weighted RAOI scenario with the equally weighted scenario to qualitatively determine how weighting factors within each RAOI scenario impacted model output. We also generated differential maps that provide insights into which MEs under the expert-weighted models had the greatest increase or decrease in RAOI score relative to the equally weighted models (i.e., a large positive change in RAOI score indicates a greater increase in RAOI score under the expert-weighted model relative to the equally weighted model). To determine the sensitivity of the RAOI to individual factors within each scenario, we: (1) sequentially removed the four factors with the highest weight in both the shellfish and seaweed RAOI scenario that incorporated all environmental, socioeconomic, and human health factors—removing each factor one at a time (Table 2), (2) re-weighted the remaining factors in both scenarios proportionally based on the weight of the removed factor, (3) re-ran both RAOI scenarios, and (4) calculated the percent change in RAOI score for each ME with removal of each factor. We evaluated the sensitivity of the RAOI to individual factors in both the expert-weighted RAOI scenario and the equally weighted scenario.

### Caveats

We utilized a suitability analysis framework to develop the RAOI, however, we recognize alternative spatial analysis approaches have merit, particularly for selection of factors and assignment of weightings (e.g., analytic hierarchy process [AHP]). Given the intended role of the RAOI to guide on-the-ground seaweed and shellfish aquaculture development projects around the world on behalf of government and NGO institutions, coupled with the importance of including factors beyond the core environmental considerations (i.e., socioeconomic and human health considerations), it was essential to utilize the expert stakeholder panel approach to guide selection and weighting of the factors considered within the RAOI. The expert stakeholder panel approach allowed for establishment of “buy-in” to the RAOI, ensured inclusion of factors relevant to guiding decisions on aquaculture development, and improved the likelihood of its utilization.

We incorporated the best available global datasets from authoritative sources within the spatial analysis. However, in compiling these datasets, data gaps were apparent for some factors for some MEs, particularly within MEs adjacent to low or lower-middle income countries. As the RAOI score in each scenario was calculated based on the cumulative sum of an ME’s score for a given factor multiplied by its associated factor-specific weight, this resulted in a bias towards identified HOMEs (i.e., high RAOI scores) corresponding with those with greater data availability. We addressed this bias in three ways: (1) we conducted the previously described sensitivity analysis to better understand the impact of the stakeholder weightings and individual factors on model output, (2) we produced a global map series that described the amount of data available for each ME that was incorporated within each RAOI scenario, and (3) we generated a map series of complementary output for the six RAOI scenarios where, if there was a data gap for a given factor within a given ME, the weights for factors where data was available were proportionally re-scaled to compute the overall RAOI score.

## Results

### Model development

The expert stakeholder group identified 16 factors considered necessary to identify high opportunity marine ecoregions (HOMEs) for shellfish and seaweed aquaculture development that were combined to calculate the ‘Restorative Aquaculture Opportunity Index’ (RAOI) across multiple scenarios (e.g., shellfish aquaculture-specific scenario inclusive of all environmental, socioeconomic, and human health factors; Tables 1 and 2). Assigned weightings for individual factors within the RAOI scenarios that incorporated all environmental, socioeconomic, and human health spatial data layers ranged from 17.44% for nutrient pollution to 2.17% for microplastics. For both of the shellfish and seaweed aquaculture-optimized scenarios that incorporated all environmental, socioeconomic, and human health factors, when summed across all factors within a given category (i.e., environmental, socioeconomic, or human health), environmental factors collectively accounted for ~40% of the overall model weight whereas socioeconomic and human health factors each accounted for ~30% of the overall model weight (Table 2).

### Model output

#### Environmental, socioeconomic, and human health factors

Environmental–Marine ecoregions (MEs) where nutrient inputs are the most elevated represent the highest opportunity locations for development of shellfish and seaweed aquaculture to provide nutrient pollution reduction benefits. High opportunity marine ecoregions (HOMEs)—those receiving the highest RAOI scores—for these benefits include those located throughout Europe and Asia, along the East and Gulf Coasts of North America, and in northern South America (continents or sub-regions are listed in descending RAOI score order; Fig 1A). HOMEs to provide alternative local seafood products and habitat benefits to mitigate the effects of excess trawl fishing pressure are located throughout Asia and Europe, the East and Gulf Coasts of North America, in southeastern South America, and in other scattered MEs throughout the world, including Alaska and New Zealand (Fig 1B). MEs in the high temperate and polar latitudes, where seawater aragonite saturation states are generally lowest, represent HOMEs for development of seaweed aquaculture to provide local ocean acidification reduction benefits (Fig 1C). HOMEs for shellfish and seaweed aquaculture to provide habitat benefits where significant habitat degradation has occurred include: West and East Coasts of North America, Europe and southern Australia to mitigate oyster reef habitat loss (Fig 1D); western South America, South Africa, Alaska, and portions of southern Australia for kelp forest habitat loss (Fig 1E); and portions of Europe, Asia, the Caribbean, and southern Australia for seagrass bed habitat loss (Fig 1F).

**Fig 2:**
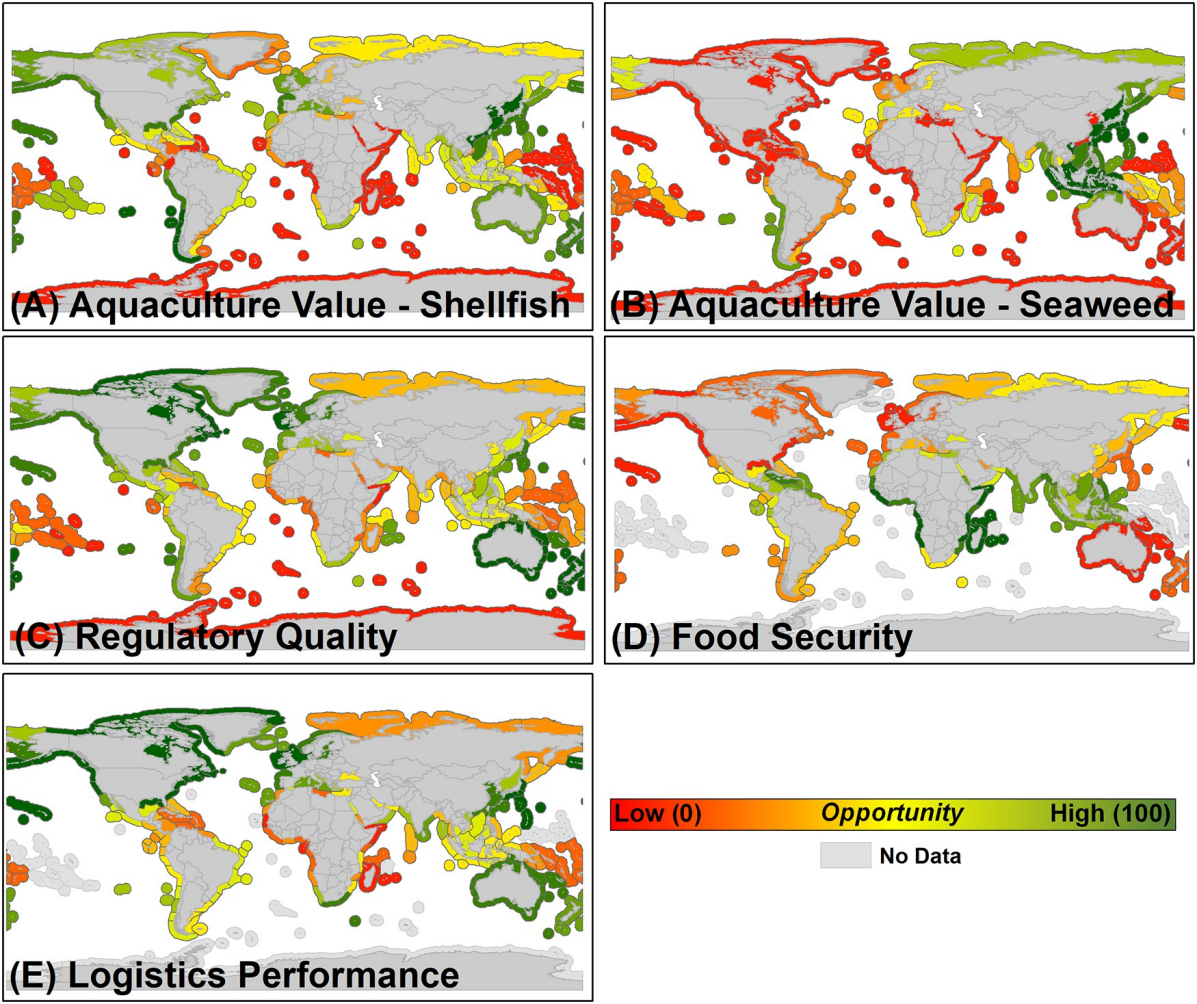
Socioeconomic factors integrated within the restorative aquaculture opportunity index used in determining high opportunity marine ecoregions for shellfish and seaweed restorative aquaculture development, including (A) aquaculture production value for shellfish, (B) aquaculture production value for seaweed, (C) regulatory quality, (D) food security, and (E) logistics performance. All factors are re-scaled to represent where shellfish and seaweed aquaculture development would provide maximum benefits. For example, marine ecoregions with the greatest regulatory quality (C) are re-scaled to represent the highest opportunity ecoregions (dark green) where shellfish and seaweed aquaculture could be adequately governed to ensure sustainable development.

Socioeconomic–Recent shellfish aquaculture production data provide an indicator of MEs where additional sustainable shellfish aquaculture industry development to provide ecosystem and societal benefits could be supported. HOMEs based on recent aquaculture production include those in Asia, western South America, portions of North America and Europe, New Zealand, and Australia (Fig 2A). Recent seaweed aquaculture production yielded HOMEs identified in Asia and western South America (Fig 2B). HOMEs based on regulatory quality include those in North America, Europe, Australia, New Zealand, western South America, and portions of Asia (Fig 2C). Food security enhancement opportunities are greatest in MEs in Africa, Asia, and the Caribbean (Fig 2D). HOMEs based on the quality of logistics performance—an indicator of infrastructure and shipment logistics capacity—are located in North America, Europe, Asia, Australia, and South Africa (Fig 2E).

**Fig 3:**
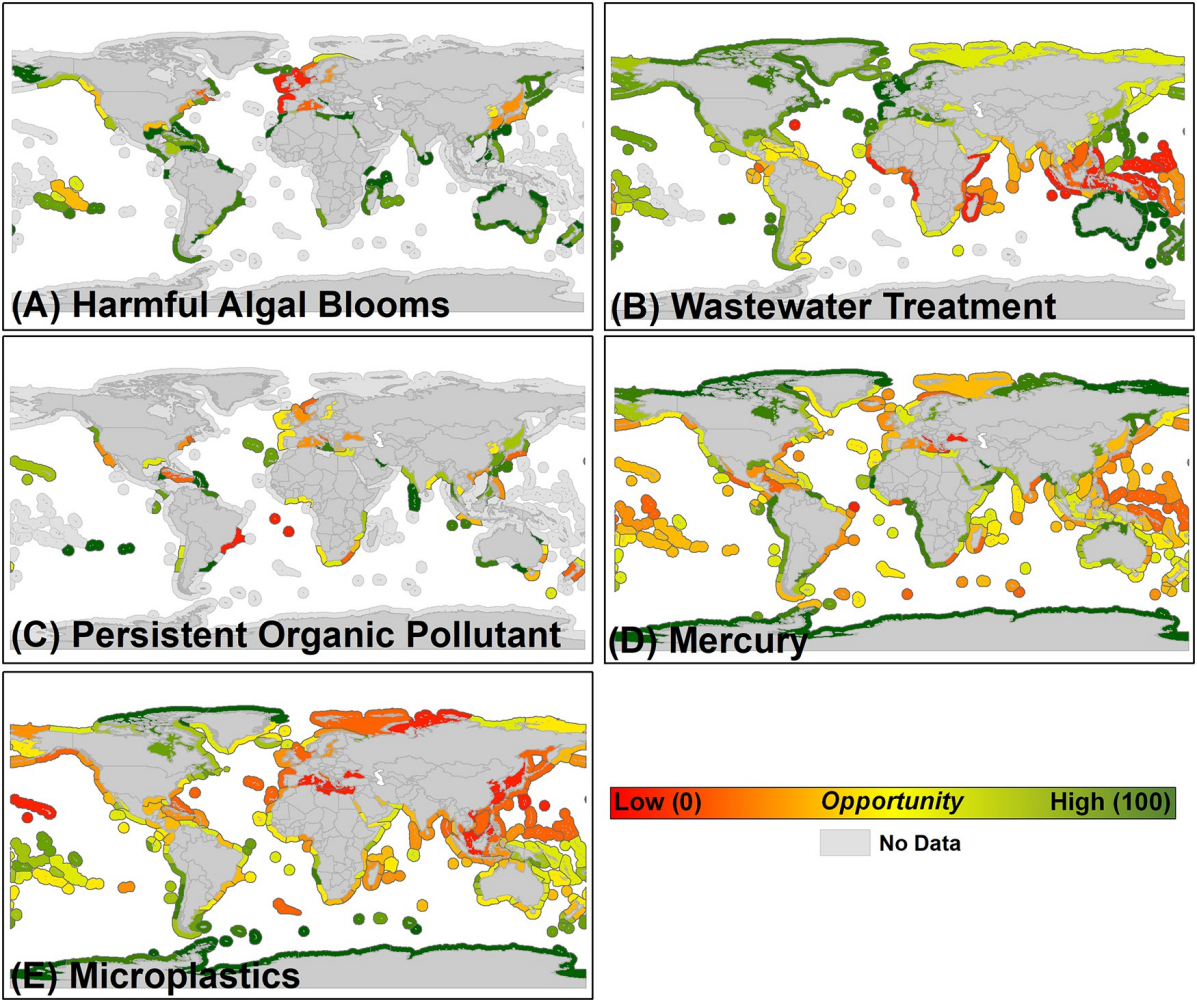
Human health factors integrated within the restorative aquaculture opportunity index used in determining high opportunity marine ecoregions for shellfish and seaweed restorative aquaculture development, including (A) harmful algal blooms, (B) wastewater treatment, (C) persistent organic pollutants, (D) mercury, and (E) microplastics. All factors are re-scaled to represent where shellfish and seaweed aquaculture development would be least likely to be impacted by these human health factors. For example, marine ecoregions with the lowest average mercury concentrations (D) are re-scaled to represent the highest opportunity ecoregions (dark green) where shellfish and seaweed aquaculture would be least likely to be impacted by elevated mercury concentrations.

Human Health–Harmful algal bloom threats are most prevalent in MEs of Europe, and parts of Asia and North America, yielding these as the lowest opportunity MEs based on the potential human health threat of harmful algal blooms (Fig 3A). MEs of central Africa, Southeast Asia, eastern South America and the Caribbean are most impacted by insufficient wastewater treatment (Fig 3B). Persistent organic pollutant threats are most pronounced in MEs of western South America, portions of Europe, the East and West Coasts of North America, and in other scattered MEs throughout the world, including southern Africa, New Zealand and portions of Asia (Fig 3C). MEs of Europe, Southeast Asia, and the East Coasts of North and South America are most threatened by mercury pollution (Fig 3D). Microplastic contamination threats are greatest throughout MEs of Europe, Asia, and North America (Fig 3E).

### Restorative Aquaculture Opportunity Index (RAOI) scenarios

**Fig 4:**
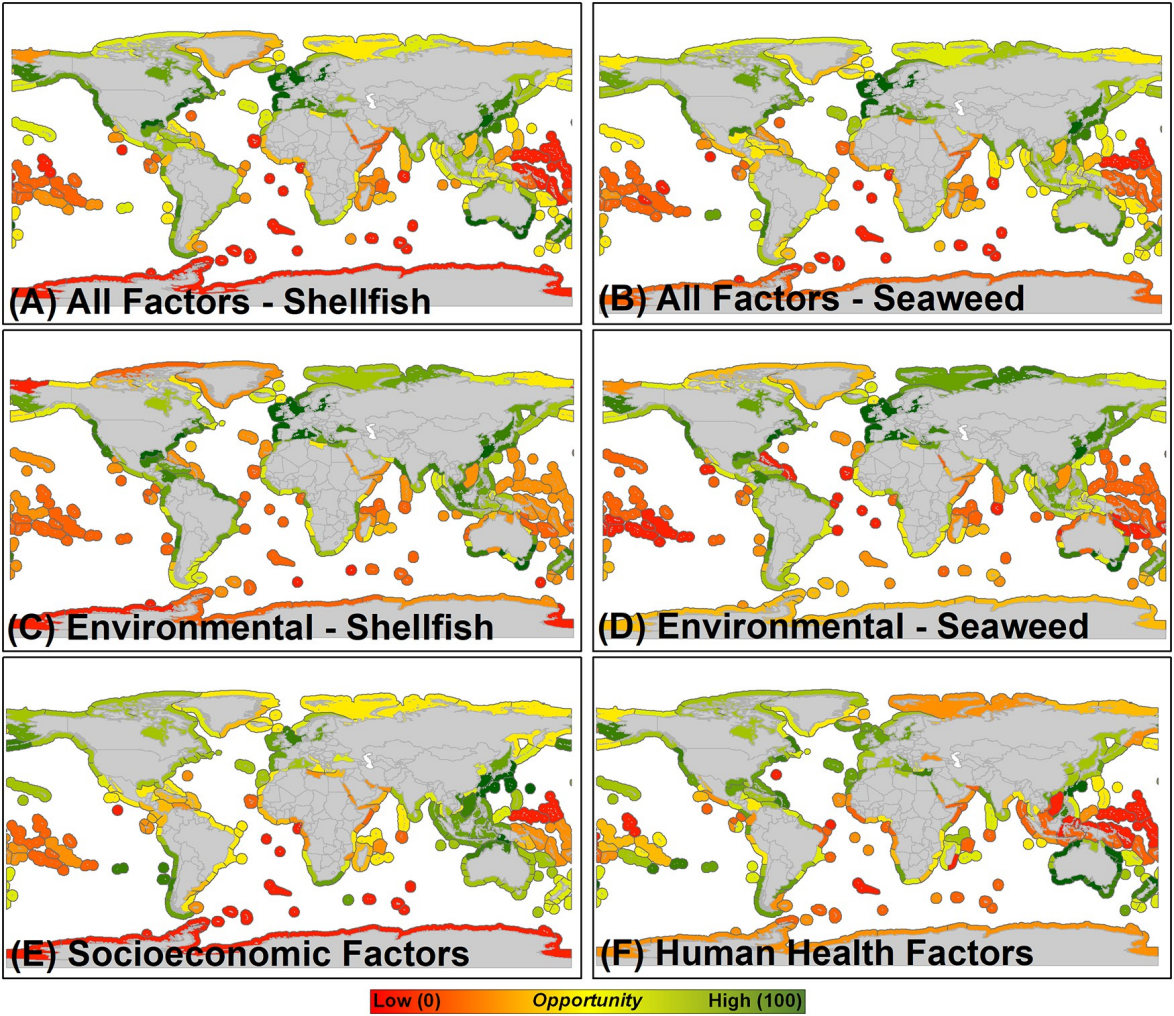
High (green) to low (red) opportunity marine ecoregions for development of (A) shellfish aquaculture and (B) seaweed aquaculture based on the synthesis of all environmental, socioeconomic, and human health factors (Table 1) according to their assigned weights (Table 2) within the restorative aquaculture opportunity index. High opportunity marine ecoregions based on the synthesis of all environmental factors only (C) and (D), socioeconomic factors only (E), and human health factors only (F) according to their assigned weights.

HOMEs for both shellfish and seaweed aquaculture development as identified in both RAOI scenarios that incorporated all environmental, socioeconomic, and human health factors are distributed throughout Europe, North and South America, Asia and Oceania (Fig 4A and 4B). The top 25 HOMEs for shellfish aquaculture development are predominantly centered on Oceania, North America, and specific portions of Asia (Table 3), whereas the top 25 HOMEs for seaweed aquaculture development are distributed throughout Europe, Asia, Oceania, and North and South America (Table 4). Within the RAOI scenario that incorporated only environmental factors for both seaweed and shellfish, HOMEs are located within Europe, North America, Asia, the Caribbean, southern Australia, and portions of South America and southern Africa (Fig 4C and 4D). Asia, Australia, Europe, western South America, southern Africa, and North America host HOMEs based on the RAOI scenario that incorporated only socioeconomic factors (Fig 4E). Human health concerns are minimized in MEs of Australia, New Zealand, North America, Europe, and portions of Asia, southern South America, and southern Africa (Fig 4F).

**Table 3:** Top 25 highest opportunity marine ecoregions for development of shellfish aquaculture from the restorative aquaculture opportunity analysis scenario where all environmental, socioeconomic, and human health factors are incorporated. The ‘Overall Composite Score’ represents the sum of the suitability scores for each factor included within the scenario multiplied by its associated weight (Table 2). For each marine ecoregion, the adjacent continent, average composite environmental, socioeconomic, and human health suitability scores, and overall composite suitability scores are presented. Dashes indicate factors for which data is unavailable for a given marine ecoregion. Data indicating full composite scores for all marine ecoregions are provided in S1 Data.

**Marine Ecoregion**	**Adjacent Continent**	*Environmental*						*Socioeconomic*				
		Nutrient Pollution Suitability	Habitat Loss (Oyster) Suitability	Habitat Loss (Kelp) Suitability	Habitat Loss (Seagrass) Suitability	Trawl Fishing Pressure Suitability	**Average Composite Environmental**	Aquaculture Value (Shellfish) Suitability	Regulatory Quality Suitability	Food Security Suitability	Logistics Performance Index Suitability	**Average Composite Socioeconomic**
North Sea	Europe	1.00	1.00	0.67	0.33	1.00	0.80	0.77	0.94	0.05	1.00	0.69
South Australian Gulfs	Australia	0.53	1.00	1.00	1.00	0.63	0.83	0.78	0.98	0.04	0.90	0.67
Bassian	Australia	0.63	1.00	0.67	0.67	0.66	0.72	0.78	0.98	0.04	0.90	0.67
Manning-Hawkesbury	Australia	0.63	1.00	0.67	0.67	0.67	0.73	0.78	0.98	0.04	0.90	0.67
Leeuwin	Australia	0.54	1.00	0.33	0.33	0.64	0.57	0.78	0.98	0.04	0.90	0.67
South European Atlantic Shelf	Europe	0.88	1.00	0.00	1.00	0.89	0.75	0.83	0.81	0.10	0.86	0.65
Northern Gulf of Mexico	North America	0.96	0.33	-	0.67	0.87	0.57	0.81	0.88	0.05	0.93	0.67
Northeastern New Zealand	Australia	0.61	0.33	0.00	0.67	0.75	0.47	0.84	0.99	0.07	0.72	0.65
Celtic Seas	Europe	0.87	0.67	0.67	-	0.93	0.63	0.77	0.94	0.01	0.97	0.67
Virginian	North America	0.86	1.00	-	0.67	0.94	0.69	0.82	0.92	0.00	0.99	0.68
Baltic Sea	Europe	0.88	0.67	-	1.00	0.79	0.67	0.58	0.87	0.11	0.88	0.61
East China Sea	Asia	0.96	-	-	1.00	0.99	0.59	0.98	0.58	0.30	0.86	0.68
Cape Howe	Australia	0.42	1.00	0.33	0.67	0.65	0.61	0.78	0.98	0.04	0.90	0.67
Gulf of Maine/Bay of Fundy	North America	0.74	-	0.67	0.67	0.89	0.59	0.81	0.93	0.02	0.98	0.68
Oregon, Washington, Vancouver Coast and Shelf	North America	0.71	0.67	0.33	-	0.80	0.50	0.81	0.92	0.02	0.98	0.68
Tweed-Moreton	Australia	0.51	1.00	-	0.00	0.68	0.44	0.78	0.98	0.04	0.90	0.67
Western Bassian	Australia	0.33	-	0.67	-	0.63	0.33	0.78	0.98	0.04	0.90	0.67
Northern California	North America	0.58	1.00	0.67	-	0.72	0.59	0.82	0.92	0.00	0.99	0.68
Carolinian	North America	0.73	0.67	-	0.33	0.81	0.51	0.82	0.92	0.00	0.99	0.68
Western Mediterranean	Europe	0.85	0.67	-	0.67	0.81	0.60	0.81	0.59	0.25	0.75	0.60
Floridian	North America	0.65	0.67	-	0.67	0.82	0.56	0.82	0.92	0.00	0.99	0.68
Central New Zealand	Australia	0.66	0.33	-	-	0.87	0.37	0.84	0.99	0.07	0.72	0.65
South Kuroshio	Asia	0.54	-	-	-	0.87	0.28	0.87	0.83	0.20	0.88	0.69
Yellow Sea	Asia	0.86	0.67	-	-	0.97	0.50	0.99	0.47	0.34	0.84	0.66
Southern California Bight	North America	0.55	1.00	0.00	0.67	0.78	0.60	0.77	0.74	0.22	0.72	0.61
**Marine Ecoregion**	*Human Health*						**Overall Composite Score**					
	Hamful Algal Blooms Suitability	Wastewater Treatment Suitability	Persistent Organic Pollutant Suitability	Mercury Suitability	Microplastics Suitability	**Average Composite Human Health**						
North Sea	0.09	0.98	0.39	0.73	0.26	0.49	**71.44**					
South Australian Gulfs	0.74	1.00	-	0.78	0.48	0.60	**69.71**					
Bassian	0.61	1.00	0.46	0.67	0.41	0.63	**69.44**					
Manning-Hawkesbury	0.68	1.00	0.37	0.53	0.48	0.61	**69.14**					
Leeuwin	0.84	1.00	0.82	0.72	0.45	0.76	**68.76**					
South European Atlantic Shelf	-	0.91	0.51	0.60	0.28	0.46	**66.57**					
Northern Gulf of Mexico	0.33	0.65	0.59	0.81	0.40	0.55	**65.52**					
Northeastern New Zealand	1.00	0.84	0.59	0.74	0.45	0.72	**65.49**					
Celtic Seas	0.08	1.00	0.53	0.55	0.33	0.50	**65.23**					
Virginian	0.24	0.69	0.34	0.39	0.41	0.42	**64.70**					
Baltic Sea	0.24	0.79	0.51	0.83	0.32	0.54	**64.38**					
East China Sea	0.24	0.35	0.68	0.58	0.11	0.39	**62.97**					
Cape Howe	0.80	1.00	-	0.65	0.46	0.58	**62.51**					
Gulf of Maine/Bay of Fundy	0.30	0.75	0.27	0.73	0.55	0.52	**62.46**					
Oregon, Washington, Vancouver Coast and Shelf	0.30	0.73	0.70	0.72	0.35	0.56	**61.90**					
Tweed-Moreton	0.77	1.00	0.50	0.55	0.47	0.66	**61.40**					
Western Bassian	0.90	1.00	1.00	0.71	0.41	0.80	**61.15**					
Northern California	0.38	0.69	0.36	0.73	0.35	0.50	**60.77**					
Carolinian	0.72	0.69	-	0.49	0.36	0.45	**59.89**					
Western Mediterranean	0.17	0.79	0.39	0.49	0.19	0.41	**59.37**					
Floridian	0.42	0.69	-	0.73	0.46	0.46	**58.95**					
Central New Zealand	0.72	0.84	0.30	0.68	0.40	0.59	**58.92**					
South Kuroshio	1.00	0.58	0.80	0.56	0.22	0.63	**58.76**					
Yellow Sea	0.40	0.27	0.49	0.79	0.00	0.39	**58.75**					
Southern California Bight	0.49	0.50	0.40	0.78	0.40	0.51	**58.28**					

**Table 4:** Top 25 highest opportunity marine ecoregions for development of seaweed aquaculture from the restorative aquaculture opportunity analysis scenario where all environmental, socioeconomic, and human health factors are incorporated. The ‘Overall Composite Score’ represents the sum of the suitability scores for each factor included within the scenario multiplied by its associated weight (Table 2). For each marine ecoregion, the adjacent continent, average composite environmental, socioeconomic, and human health suitability scores, and overall composite suitability scores are presented. Dashes indicate factors for which data is unavailable for a given marine ecoregion. Data indicating full composite scores for all marine ecoregions are provided in S1 Data.

**Marine Ecoregion**	**Adjacent Continent**	*Environmental*						*Socioeconomic*				
		Nutrient Pollution Suitability	Habitat Loss (Oyster) Suitability	Habitat Loss (Kelp) Suitability	Habitat Loss (Seagrass) Suitability	Trawl Fishing Pressure Suitability	**Overall Composite Environmental**	Aquaculture Value (Seaweed) Suitability	Regulatory Quality Suitability	Food Security Suitability	Logistics Performance Index Suitability	**Overall Composite Socioeconomic**
North Sea	Europe	1.00	1.00	0.67	0.33	1.00	0.80	0.50	0.94	0.05	1.00	0.62
Baltic Sea	Europe	0.88	0.67	-	1.00	0.79	0.67	0.58	0.87	0.11	0.88	0.61
South European Atlantic Shelf	Europe	0.88	1.00	0.00	1.00	0.89	0.75	0.61	0.81	0.10	0.86	0.59
Celtic Seas	Europe	0.87	0.67	0.67	-	0.93	0.63	0.49	0.94	0.01	0.97	0.60
East China Sea	Asia	0.96	-	-	1.00	0.99	0.59	1.00	0.58	0.30	0.86	0.69
Northeastern Honshu	Asia	0.71	-	-	-	0.82	0.31	0.97	0.90	0.09	0.98	0.74
Bassian	Australia	0.63	1.00	0.67	0.67	0.66	0.72	0.00	0.98	0.04	0.90	0.48
Leeuwin	Australia	0.54	1.00	0.33	0.33	0.64	0.57	0.00	0.98	0.04	0.90	0.48
Central Chile	South America	0.49	0.67	1.00	-	0.74	0.58	0.78	0.75	0.21	0.62	0.59
Western Mediterranean	Europe	0.85	0.67	-	0.67	0.81	0.60	0.58	0.59	0.25	0.75	0.54
Manning-Hawkesbury	Australia	0.63	1.00	0.67	0.67	0.67	0.73	0.00	0.98	0.04	0.90	0.48
Oregon, Washington, Vancouver Coast	North America	0.71	0.67	0.33	-	0.80	0.50	0.00	0.92	0.02	0.98	0.48
Central Kuroshio Current	Asia	0.86	-	-	-	0.90	0.35	0.97	0.90	0.09	0.98	0.74
Sea of Japan/East Sea	Asia	0.89	-	-	0.33	0.92	0.43	0.96	0.51	0.26	0.64	0.59
Araucanian	South America	0.74	-	-	-	0.80	0.31	0.78	0.80	0.20	0.63	0.60
South Australian Gulfs	Australia	0.53	1.00	1.00	1.00	0.63	0.83	0.00	0.98	0.04	0.90	0.48
Northern Gulf of Mexico	North America	0.96	0.33	-	0.67	0.87	0.57	0.00	0.88	0.05	0.93	0.47
South Kuroshio	Asia	0.54	-	-	-	0.87	0.28	0.96	0.83	0.20	0.88	0.72
Gulf of Maine/Bay of Fundy	North America	0.74	-	0.67	0.67	0.89	0.59	0.00	0.93	0.02	0.98	0.48
Virginian	North America	0.86	1.00	-	0.67	0.94	0.69	0.00	0.92	0.00	0.99	0.48
Southern Norway	Europe	0.75	-	0.67	-	0.89	0.46	0.00	0.93	0.07	0.89	0.47
Western Bassian	Australia	0.33	-	0.67	-	0.63	0.33	0.00	0.98	0.04	0.90	0.48
Northeastern New Zealand	Australia	0.61	0.33	0.00	0.67	0.75	0.47	0.00	0.99	0.07	0.72	0.45
Malacca Strait	Asia	0.83	-	-	-	0.87	0.34	0.96	0.60	0.49	0.61	0.67
Northern California	North America	0.58	1.00	0.67	-	0.72	0.59	0.00	0.92	0.00	0.99	0.48
**Marine Ecoregion**	*Human Health*											
	Wastewater Treatment Suitability	Persistent Organic Pollutant Suitability	Mercury Suitability	**Overall Composite Human Health**	**Overall Composite Score**							
North Sea	0.98	0.39	0.73	0.70	73.75							
Baltic Sea	0.79	0.51	0.83	0.71	69.27							
South European Atlantic Shelf	0.91	0.51	0.60	0.67	68.15							
Celtic Seas	1.00	0.53	0.55	0.70	66.88							
East China Sea	0.35	0.68	0.58	0.54	63.72							
Northeastern Honshu	0.77	0.67	0.54	0.66	62.02							
Bassian	1.00	0.46	0.67	0.71	61.13							
Leeuwin	1.00	0.82	0.72	0.85	59.73							
Central Chile	0.58	0.60	0.84	0.67	59.29							
Western Mediterranean	0.79	0.39	0.49	0.56	58.92							
Manning-Hawkesbury	1.00	0.37	0.53	0.63	58.30							
Oregon, Washington, Vancouver Coast	0.73	0.70	0.72	0.72	58.24							
Central Kuroshio Current	0.77	0.31	0.41	0.50	58.17							
Sea of Japan/East Sea	0.38	0.63	0.57	0.53	57.52							
Araucanian	0.63	0.62	0.79	0.68	57.37							
South Australian Gulfs	1.00	-	0.78	0.59	57.22							
Northern Gulf of Mexico	0.65	0.59	0.81	0.68	56.67							
South Kuroshio	0.58	0.80	0.56	0.65	56.07							
Gulf of Maine/Bay of Fundy	0.75	0.27	0.73	0.58	55.36							
Virginian	0.69	0.34	0.39	0.47	54.97							
Southern Norway	0.84	0.26	0.66	0.59	54.38							
Western Bassian	1.00	1.00	0.71	0.90	54.30							
Northeastern New Zealand	0.84	0.59	0.74	0.72	53.67							
Malacca Strait	0.04	0.67	0.70	0.47	52.61							
Northern California	0.69	0.36	0.73	0.59	52.06							

### Model sensitivity

**Fig 5:**
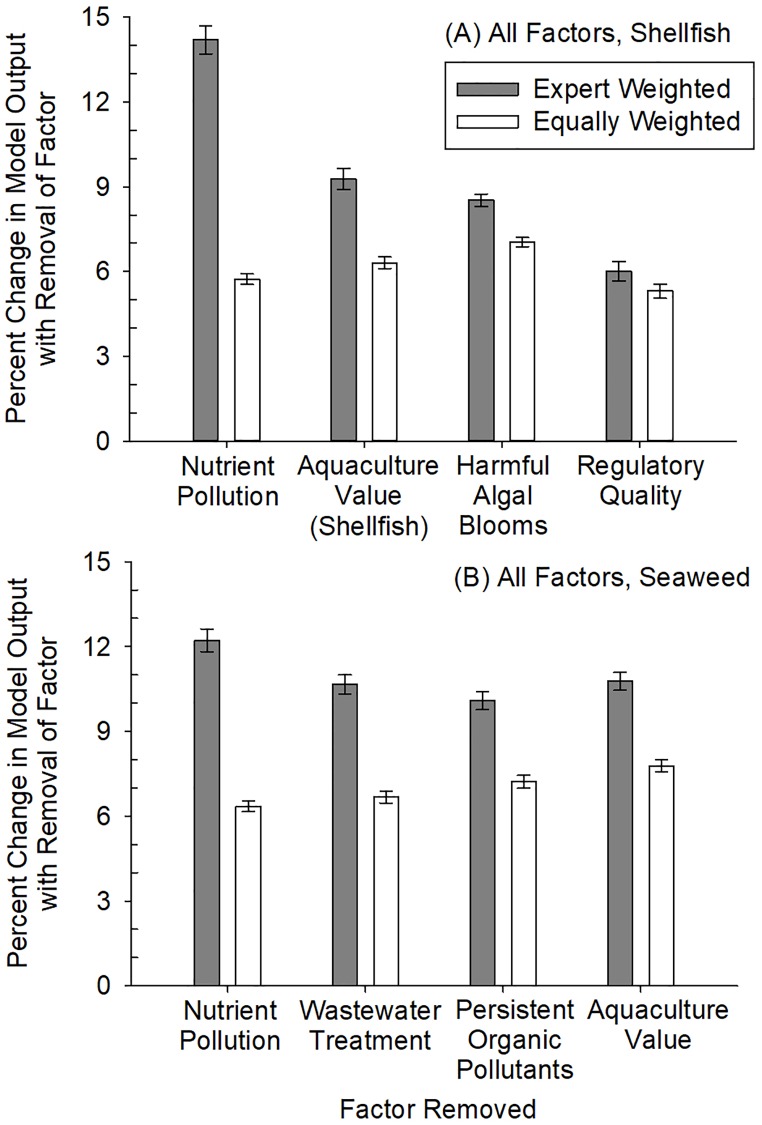
Model sensitivity analysis for (A) shellfish aquaculture RAOI scenario with all environmental, socioeconomic, and human heath factors and (B) seaweed aquaculture RAOI scenario with all factors where: (1) the four factors with the highest weights (stakeholder, expert-weighted RAOI model) were individually removed, (2) the remaining factors were proportionally re-weighted based on the weight of the removed factor, and (3) the percent change in model output was calculated on an marine ecoregion-by-marine ecoregion basis for removal of each factor. The same process was followed in the equally weighted model.

The stakeholder, expert-weighted models for both the shellfish and seaweed aquaculture RAOI scenarios were generally most sensitive to the factors with higher weightings. For example, the expert-weighted RAOI scenario for shellfish aquaculture that incorporated all environmental, socioeconomic, and human health factors, the percent change in RAOI averaged among all grid cells was 14.2% ± 0.5% SE with the removal of the nutrient pollution factor (weighted at 17.44%) and 9.3% ± 0.4% SE with removal of the aquaculture value (shellfish) factor (weighted at 10.63%; Fig 5A). The equally weighted RAOI scenario for shellfish aquaculture that incorporated all factors was most sensitive to the removal of the harmful algal blooms factor, followed by aquaculture value (shellfish), nutrient pollution, and regulatory quality. The expert-weighted RAOI scenario for seaweed aquaculture that incorporated all factors was most sensitive to the nutrient pollution factor (12.2% ± 0.4% SE; weighted at 14.69%; Fig 5B) followed by the aquaculture value (seaweed) factor (10.8% ± 0.3% SE; weighted at 10.63%). The equally weighted scenario for seaweed aquaculture that incorporated all factors was most sensitive to the removal of the aquaculture value (seaweed) factor, followed by persistent organic pollutants, wastewater treatment, and nutrient pollution.

**Fig 6:**
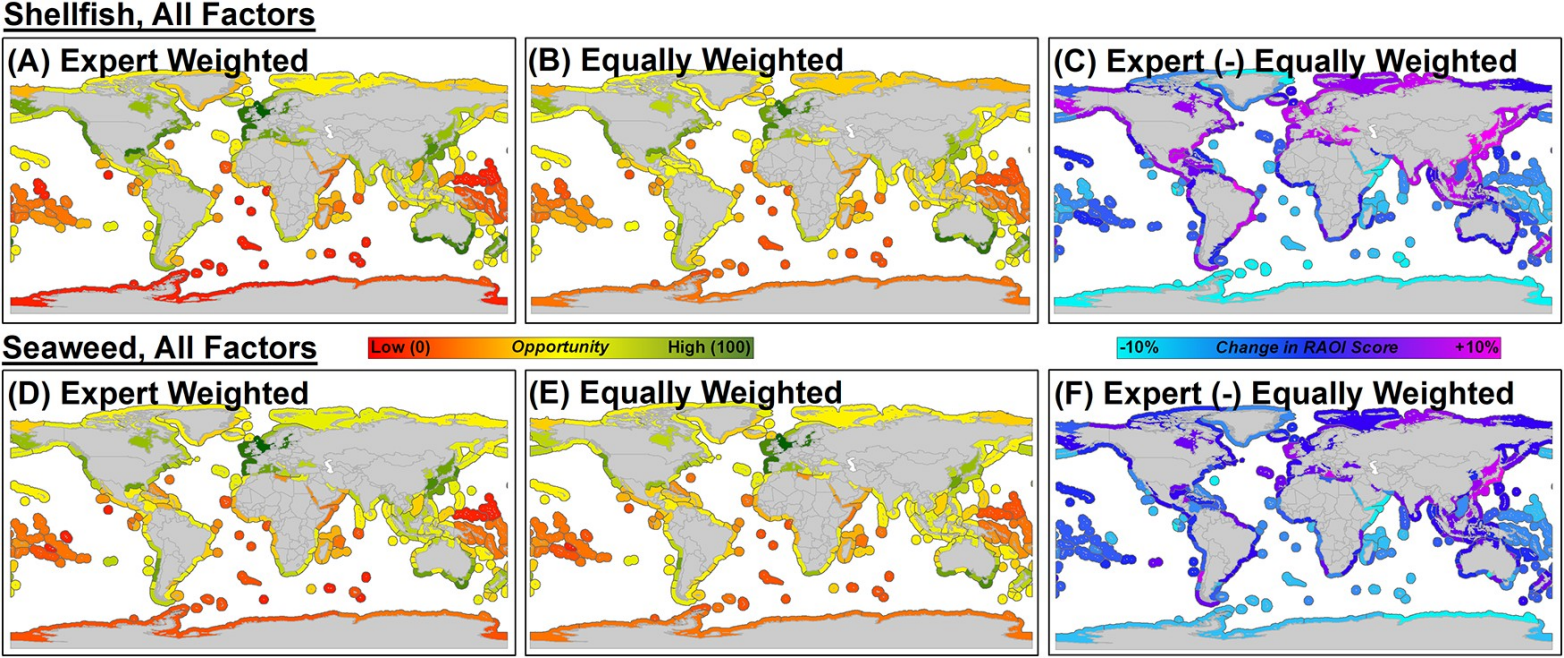
Comparison of identified high (green) to low (red) opportunity marine ecoregions between the stakeholder, expert-weighted models that incorporated all environmental, socioeconomic, and human health factors for shellfish (A) and seaweed (D), and the equally weighted models that incorporated all factors for shellfish (B) and seaweed (E). The differential maps for shellfish (C) and seaweed (F) represent the subtraction of the RAOI scores for the equally weighted models from the expert-weighted models. For example, marine ecoregions with changes in RAOI score close to +10% (magenta) represent those with the greatest increase in RAOI score under the expert-weighted model relative to the equally weighted model. Factors included in each scenario and associated weightings are provided in Table 2.

For both RAOI scenarios for shellfish and seaweed aquaculture that incorporated all factors, the equally weighted models were less selective than the expert-weighted models (Fig 6). The equally weighted models were characterized by a distribution of RAOI scores centered around a moderate, average RAOI score whereas the expert-weighted models exhibited RAOI scores with greater differentiation of high, moderate and low RAOI scores amongst MEs (S3 Fig). The differential maps (Fig 6C and 6F) indicate which MEs under the expert-weighted models had the greatest increase or decrease in RAOI score relative to the equally weighted models (i.e., a large positive change in RAOI score indicates a greater increase in RAOI score under the expert-weighted model relative to the equally weighted model). MEs of Asia and portions of Europe, South and North America, and New Zealand experienced the greatest increase in RAOI score under the expert-weighted RAOI scenario for shellfish aquaculture that incorporated all factors relative to the equally weighted model. MEs of portions of Asia, Europe, and South America experienced the greatest increase in RAOI score under the expert-weighted RAOI scenario for seaweed aquaculture that incorporated all factors relative to the equally weighted model.

### Limitations

We recognize that data limitations did impact the RAOI scores for MEs within the analysis and addressed this in multiple ways. Limitations in environmental data for MEs associated with Southeast Asia, Africa, and many island nations around the world reduced the associated RAOI scores for these MEs (Fig 4), and we provide a summary of the amount of data available by ME that was used to compute the RAOI in the six restorative aquaculture opportunity analysis scenarios in S1 Fig. For example, insufficient data exists to describe the status of critical coastal habitats such as oyster reefs, kelp forests, and seagrass beds within these MEs (Fig 1, S1 Fig). There is a notable lack of data regarding food security status and logistics performance capacity for many Pacific Island nations (Fig 2, S1 Fig), even though food security challenges and global market opportunities do exist within these nations [32] Information derived from the summary of data availability (S1 Fig) can be used to assess confidence in the assigned RAOI score for various MEs. For example, MEs of Europe were generally assigned high RAOI scores (Fig 4) and have high amounts of data available that were used to compute the RAOI, implying that there is a high degree of confidence in these as HOMEs for development of shellfish and seaweed aquaculture to provide environmental and socioeconomic benefits. In other cases, MEs with higher amounts of data available (e.g., those of southern Africa) were identified as moderate or lower opportunity. Another approach taken to address the impact of data limitations on RAOI score was to generate a map series of complementary output for the six RAOI scenarios where, if for a given factor for a given ME there was a data gap, the weights for factors where data was available were proportionally re-scaled to compute the overall RAOI score (S2 Fig). These proportionally re-scaled scenarios served to normalize the score of MEs for which less data was available—in many cases, this served to increase the RAOI score of MEs for which limited data was available, but the available data indicated a greater degree of opportunity (e.g., the ‘Exmouth to Broome’ ME adjacent to Australia, S1 Table). While these scenarios generally provide insight into promising MEs for shellfish and seaweed aquaculture to provide societal benefits, the proportional re-scaling approach tended towards assigning higher RAOI scores to MEs for which limited environmental data were available. In general, the HOMEs identified within these scenarios appear to generally have sufficient socioeconomic enabling conditions, but the environmental challenge for shellfish and seaweed aquaculture to combat and in some cases the level of human health concern is less known, hence our focus throughout the paper on the non-rescaled scenarios (i.e., Tables 3 and 4, Fig 4 and 4B).

## Discussion

As one of the fastest growing forms of primary food production valued at US$243.5B globally, aquaculture has become a significant part of global food systems and agribusiness [2]. Aligned with this growth trend, governments, international development organizations, and investors around the world are embarking on new efforts to increase development of the industry with the objectives of creating jobs, improving human nutrition, and bolstering food security. However, given the negative ecological consequences associated with aquaculture when inappropriately developed (e.g., environmentally destructive practices, poorly sited operations), significant management attention is often placed on addressing these impacts [6]. More rarely is the potential for localized environmental improvement through ecosystem service provision (e.g., nutrient pollution mitigation benefits associated with shellfish aquaculture) identified as significant rationale or driver for new aquaculture development efforts. Our global-scale spatial analysis identified marine ecoregions of significant environmental, socioeconomic, and human health opportunity that should be the focus of targeted efforts to drive change in aquaculture policy, capacity-building, and industry development. The current global distribution of shellfish and seaweed aquaculture operations, the magnitude of global production (US$40.9B and 47.2M tonnes) [2], and the annual global growth rate of the overall aquaculture sector (5.8% per year in 2016), suggest that there is a significant opportunity for policy and other interventions to shift the sector’s trajectory towards one that maximizes ecosystem and social benefits. The present study sought to fill an existing void in our understanding of where the ecosystem recovery and societal benefits are most likely to be realized through shellfish and seaweed aquaculture development.

High opportunity marine ecoregions (HOMEs) distributed throughout Europe, North and South America, Asia, and Oceania were identified as having the highest Restorative Aquaculture Opportunity Index (RAOI) scores within the scenarios that incorporated all environmental, socioeconomic, and human health factors—indicating the substantial opportunity across the globe for shellfish and seaweed aquaculture to provide environmental and socioeconomic benefits (Fig 4A and 4B, Tables 3 and 4). Within identified HOMEs, the environmental opportunity is generally greatest (i.e., potential to mitigate nutrient pollution, trawl fishing pressure, ocean acidification risk, habitat loss; Fig 1 and see ‘Rationale’ provided in Table 1), socioeconomic conditions are generally sufficient (i.e., history of aquaculture production, regulatory quality, logistics performance; Fig 2), and human health concerns are generally minimized (e.g., sufficient wastewater treatment; Fig 3). A good example is the ‘North Sea’ marine ecoregion (ME), which was identified as having the highest opportunity for both shellfish and seaweed aquaculture (RAOI score of 71.44 for shellfish aquaculture, 73.75 for seaweed Aquaculture; Tables 3 and 4). Within the North Sea, nutrient inputs, habitat loss, and trawl fishing pressure are elevated yielding a substantial environmental challenge and opportunity for shellfish and seaweed aquaculture to combat; historic aquaculture production, regulatory quality and logistics performance provide enabling socioeconomic conditions within the ME. While wastewater treatment is sufficient and mercury pollution concerns are minimal, a history of harmful algal blooms, high microplastic concentrations, and elevated persistent organic pollutant concentrations yield a marginal overall human health factors score for the North Sea. As RAOI scores represent a composite across environmental, socioeconomic, and human health factors, no single ME received an RAOI score of 100 indicative of the counterbalance of opportunity and risk associated with shellfish and seaweed aquaculture development. Notably, HOMEs tended to be adjacent to upper-middle or high income nations; as described further below, these results do not preclude the importance of sustainable aquaculture development within MEs adjacent to low or lower-middle income nations.

Of the top 25 HOMEs for shellfish aquaculture, 9 were located in Oceania (53% of all MEs in Oceania), 8 in North America (24%), 5 in Europe (28%), and 3 in Asia (7%; Table 3). For seaweed aquaculture, the top 25 HOMEs included 6 located in Europe (33% of all MEs), 6 in Asia (14%), 6 in Oceania (35%), 5 in North America (15%), and 2 in South America (8%; Table 4). Correspondingly, significant industry developments are underway within the countries adjacent to these MEs. For example, Australia’s strong enabling conditions (e.g., sound sector governance, logistics quality) have prompted development of a diverse and widespread shellfish aquaculture industry, with license holders farming a range of species in many small or isolated towns along the entirety of the country’s coastline [33]. Widespread historical commercial fishing is being recalled as an important pastime, with contemporary aquaculture put forward as an important means to increase sustainable production, refocus commercial efforts on native species, and to positively affect restoration efforts [34]. In Norway, the amount of area permitted for seaweed cultivation in the waters within and adjacent to the North Sea has tripled between 2014 and 2016 [35]. This rapid growth has been partially attributed to recognition of the nutrient recovery and bioremediation capacity of seaweeds, alongside the growing interest in development of aquaculture more broadly throughout Europe as part of the European Union’s ‘Blue Growth’ strategy. As many of the identified HOMEs correspond with areas where significant existing shellfish and seaweed aquaculture operations are ongoing, these locations represent significant opportunities for improvements in management of existing operations, siting of new operations to maximize associated benefits, and potential industry growth to achieve benefits at-scale. Given the scale at which shellfish and seaweed aquaculture operations exist within some of these MEs (e.g., China is the world’s largest aquaculture producer of shellfish) [2], even minor steps towards these suggested ecosystem-oriented improvements are likely to yield substantial benefits.

Moderate opportunity MEs were located throughout South America, Asia, and Africa, driven largely by modest environmental and socioeconomic opportunity, and some degree of human health concerns. In some cases, the RAOI scores of MEs with identified elevated environmental opportunity scores (Fig 4C and 4D) were reduced by diminished socioeconomic opportunity scores (Fig 4E)—for example, the ‘Gulf of Guinea South’ ME off the coast of Gabon and Democratic Republic of Congo. Lower opportunity MEs were focused in Southeast Asia, Africa, and the Middle East (Fig 4A and 4B), and the lower RAOI scores associated with these MEs were driven by a combination of reduced environmental and/or socioeconomic opportunity paired with human health concerns. Importantly, the results of this analysis may be limited in defining the opportunity, and potentially the importance, of sustainable aquaculture development within MEs surrounding low or lower-middle income nations. The moderate opportunity identified for MEs adjacent to these nations highlights the need to better understand the underlying challenges to ensure solutions can be effective and sustainable in the long-term. For example, while the environmental opportunity (e.g., potential to mitigate nutrient pollution and provide habitat) in MEs adjacent to India and Bangladesh is significant, challenges with insufficient regulatory quality, logistics performance, and/or human health concerns reduced the RAOI scores for these MEs and indicate the importance of broader efforts to improve governance and to address other core social challenges. Additionally, as described in greater detail within ‘Results: Limitations,’ a notable lack of data exists for MEs associated with Southeast Asia, Africa, and many island nations. We suggest prioritization of inventories of coastal habitats (e.g., oyster reefs, kelp forests, seagrass beds) and improvements in estimation of metrics of key socioeconomic parameters (e.g., food security, logistics performance) within these MEs as a management priority. Given these data limitations, we further recommend more detailed attention and consideration be given to assessing the potential for development of shellfish and seaweed aquaculture sectors in these areas.

Increasingly, evidence indicates that where aquaculture development is intentionally designed to support beneficial environmental or social outcomes, positive impacts can be returned for both people and the ecosystem [9]. Spatial planning at local, regional, national, and/or global scales can improve the environmental and social performance of aquaculture. We focused on spatial planning at a global scale to resolve marine ecoregions where targeted development of shellfish and seaweed aquaculture could provide benefits beyond food production, such as novel coastal business and ecosystem recovery opportunities. The global scale mapping products and information provided by this study can be used to guide aquaculture development projects and have implications for the establishment of policies to support sustainable aquaculture development to provide economic, social, and ecological benefits. At regional and local scales, mapping and identifying areas of existing ocean uses (e.g., shipping lanes), potential negative environmental interactions (e.g., marine mammal migration corridors), and known human health risks (e.g., wastewater outfalls, known areas of frequent harmful algal blooms) are essential to minimize social, environmental, and health risk and conflict while promoting delivery of potential ecosystem services.

Development of shellfish and seaweed aquaculture within MEs with high RAOI scores does not guarantee provision of ecosystem services or that ecosystem function will be recovered. While the present study provides valuable insight at the global-scale to guide strategic aquaculture development initiatives at the scale of MEs, ultimately, multiple factors at successive geographic and ecosystem scales will affect the extent of ecosystem delivery of aquaculture and whether farms ultimately improve or degrade ecosystem function. These factors include the functional traits of culture species, abiotic and biotic characteristics of the surrounding environment, farm-design, and farming practices [36]. For example, when unsustainably developed and/or managed, intensive water column culture of bivalve shellfish beyond a water body’s carrying capacity could generate sufficient pseudofecal and fecal carbon loading to yield localized benthic hypoxia [37].

The pre-existing extent of ecosystem degradation in a given location can make developing commercial shellfish and seaweed aquaculture for ecosystem recovery purposes more or less appealing (e.g. degraded species or areas can be subject to protective regulation). However, these areas can also provide critical opportunities to accelerate ecosystem recovery efforts through development of sustained aquaculture production to support delivery of ecosystem services, and the results of this study inform identification of regions where these opportunities are most likely to be realized. Appropriately located through use of regional and local scale spatial planning and management approaches, shellfish and seaweed aquaculture development can play a complementary role to aide governments and communities in achieving conservation, ecosystem recovery or other environmental goals. For example, within the Chesapeake Bay region, the Maryland Department of Natural Resources developed web-accessible, dynamic maps of priority areas for various forms of oyster mariculture to guide management decisions regarding where establishment of leases would most likely yield optimal oyster growth alongside water filtration ecosystem services and while minimizing the likelihood of space use conflict [38].

Concurrent with the rapid, production-centered development of commercial shellfish and seaweed aquaculture sectors, there exists nascent interest in the development of shellfish and seaweed aquaculture for bioremediation, environmental improvement, and other non-consumptive purposes [11, 39]. The RAOI scenarios focused solely on inclusion of environmental factors (i.e., Fig 4C and 4D), and the HOMEs identified at the factor-specific-level (e.g., nutrient pollution in Fig 1A) provide insight into where development of shellfish and seaweed aquaculture for these purposes is likely to be most impactful. Within these HOMEs, establishment of ‘payment for ecosystem services’ programs and/or associated policy development could shift the trajectory of the sector towards encouraging development of certain types of aquaculture, such as shellfish and seaweed aquaculture, that can provide positive environmental benefits. The enhanced recognition of where environmental and ecosystem service benefits are likely to be realized as identified within this study could provide an informative tool for prioritization of deployment of “impact”-driven investment capital into the seaweed and shellfish aquaculture sectors. In the United States, local and state governments in coastal towns throughout Cape Cod, Massachusetts are considering multiple nature-based solutions (including shellfish aquaculture) that have potential to improve nitrogen removal and achievement of U.S. Clean Water Act-mandated nutrient ‘Total Maximum Daily Load’ (TMDL) [40]. This includes investigating the potential for various financing vehicles (e.g., shellfish nitrogen removal credits) to fund a range of as many as ~40 types of nature-based solutions that can yield nitrogen removal benefits.

Through conducting this global-scale spatial analysis, we identified HOMEs for development of shellfish and seaweed aquaculture to support ecosystem recovery and benefit society. These locations of high opportunity, depending on specific environmental, socioeconomic and human health conditions, may benefit from targeted efforts to build in-region technical capacity for shellfish and seaweed aquaculture, improve sector governance, and improve environmental and economic performance of aquaculture to yield benefits to coastal ecosystems and communities. Examples of these targeted efforts include those in Indonesia and Belize where The Nature Conservancy is working with local fishers, government agencies, and NGO partners to build capacity for sustainable seaweed aquaculture sectors [41]. This includes: (1) efforts to train fishers to farm seaweed sustainably (i.e., communication of best management practices for seaweed farming through written publications [42] and in-person trainings), (2) monitoring of pilot seaweed farms to examine and quantify potential benefits and impacts of seaweed farming on water quality and local biota, (3) coordination with government agencies to establish a spatial management framework and permitting pathway for regulating seaweed farming, and (4) collaborations with major seaweed purchasers to establish incentives for suppliers employing sustainable practices.

As coastal ecosystems and communities face mounting threats, a more integrated, pragmatic, and market-driven approach to ecosystem recovery and management efforts is increasingly critical. Shellfish and seaweed aquaculture hold great promise as a tool that can play a role in improving the outcomes of these efforts when developed responsibly. The high opportunity marine ecoregions identified in this study represent those areas where targeted governance support and business development efforts by governments, international development organizations, and investors may hold the greatest promise to develop these sectors in a manner that supports positive outcomes for both ecosystems and society.

## Supporting information

S1 Fig Amount of data available by marine ecoregion utilized to compute the restorative aquaculture opportunity index in the six restorative aquaculture opportunity analysis scenarios: (A) shellfish aquaculture inclusive of all environmental, socioeconomic, and human health factors and (B) seaweed aquaculture inclusive of all factors, (C) and (D) environmental factors only, (E) socioeconomic factors only, and (F) human health factors only. Dark blue colors indicate marine ecoregions with high amounts of available data to represent factors considered within each spatial analysis scenario.
(TIFF)

S2 Fig High (green) to low (red) opportunity marine ecoregions for development of (A) shellfish aquaculture and (B) seaweed aquaculture based on the synthesis of all environmental, socioeconomic, and human health factors (Table 1) according to their assigned weights (Table 2) and reweighted based on data availability within the restorative aquaculture opportunity index. For ecoregions where data was not available for certain factors included within a given restorative aquaculture opportunity analysis scenario (Figs 1–3 and Table 2), the weightings assigned to included factors for which data was available were proportionally adjusted to sum to 100. High opportunity marine ecoregions based on the restorative aquaculture opportunity index scenario that included a synthesis of all environmental factors only is presented in panels (C) and (D), socioeconomic factors only (E), and human health factors only (F) according to their assigned weights.
(TIFF)

S3 Fig Histograms of RAOI scores associated with the expert weighted scenarios for shellfish and seaweed (A and C), and the equally weighted scenarios for shellfish and seaweed (B and D). The mean and standard deviation of RAOI scores in each scenario are included.
(TIF)

S1 Table Top 25 highest opportunity marine ecoregions for development of shellfish aquaculture from the restorative aquaculture opportunity analysis scenario where all environmental, socioeconomic, and human health factors are incorporated (Table 2) and reweighted based on data availability within the restorative aquaculture opportunity index. For ecoregions where data was not available for certain factors included within a given restorative aquaculture opportunity analysis scenario (Figs 1–3 and Table 2), the weightings assigned to included factors for which data was available were proportionally adjusted to sum to 100. Dashes indicate factors for which data is unavailable for a given marine ecoregion. Data indicating full composite scores for all marine ecoregions are provided in S1 Data.
(XLSX)

S1 Data Full composite RAOI scores for all marine ecoregions in the expert weighted shellfish and expert weighted seaweed RAOI scenarios, including the scenarios where, if there was a data gap for a given factor within a given ME, the weights for factors where data was available were proportionally re-scaled to compute the overall RAOI score (referred to in the dataset as the ‘reweighted’ scenarios). (XLSX)
