# Supplementary figures and images for: A global spatial analysis reveals where marine aquaculture can benefit nature and people
Source: PLoS One. 2019 Oct 9;14(10):e0222282. doi: 10.1371/journal.pone.0222282 (PMC6784979; doi:10.1371/journal.pone.0222282)

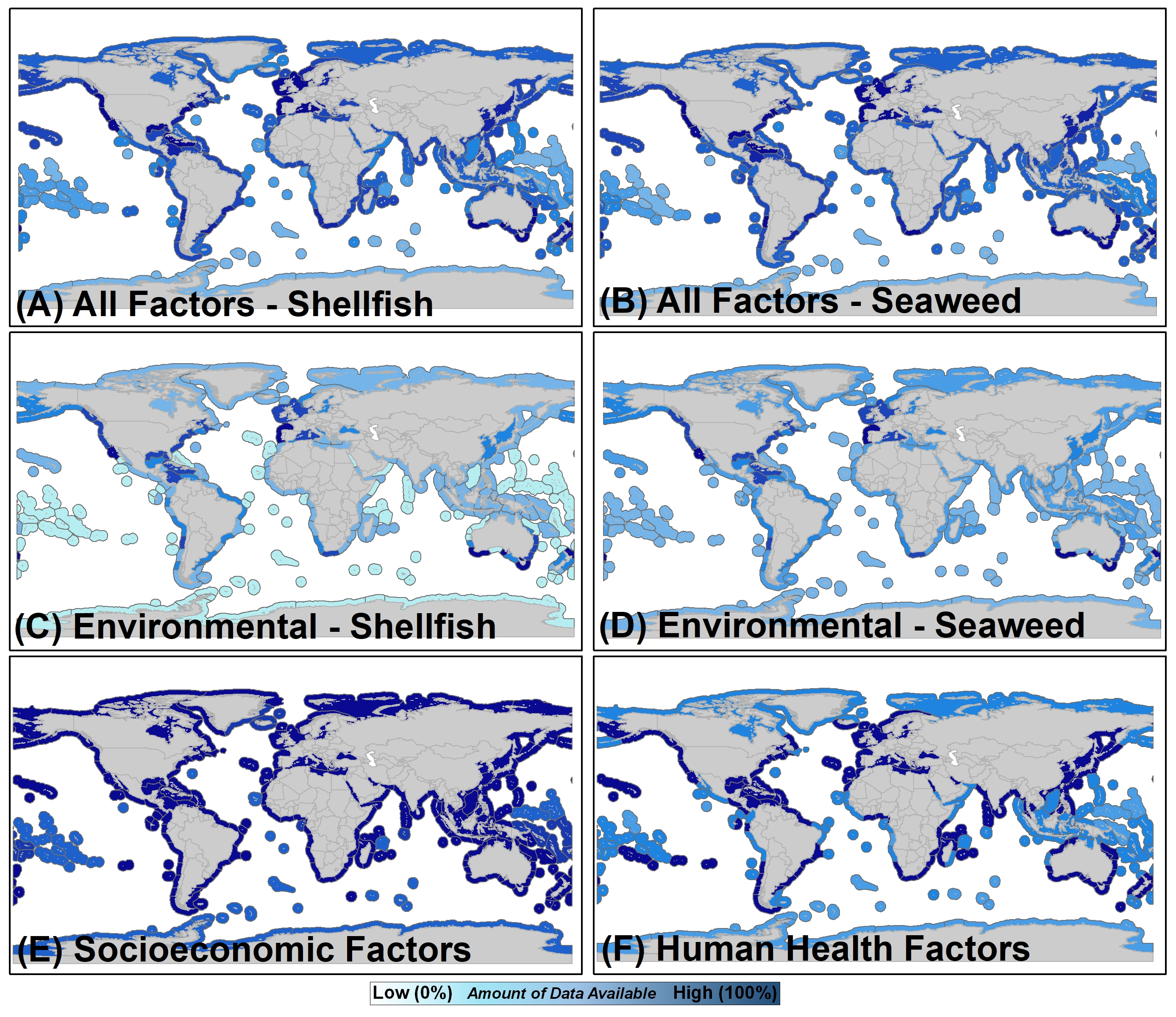

Supplement: S1 Fig — Dark blue colors indicate marine ecoregions with high amounts of available data to represent factors considered within each spatial analysis scenario. (TIFF) [file pone.0222282.s001.tiff]

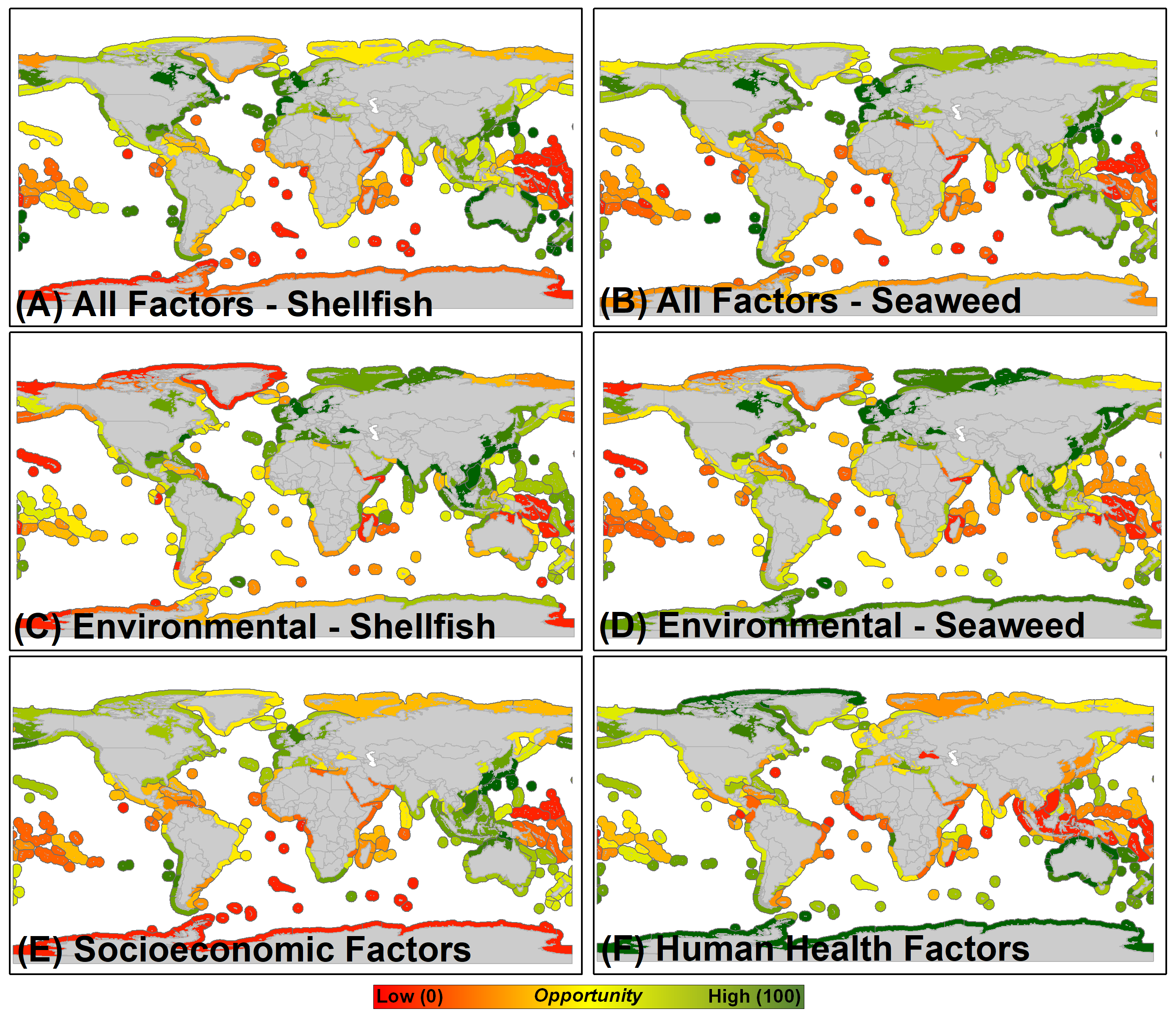

Supplement: S2 Fig — High (green) to low (red) opportunity marine ecoregions for development of (A) shellfish aquaculture and (B) seaweed aquaculture based on the synthesis of all environmental, socioeconomic, and human health factors (Table 1) according to their assigned weights (Table 2) and reweighted based on data availability within the restorative aquaculture opportunity index. For ecoregions where data was not available for certain factors included within a given restorative aquaculture opportunity analysis scenario (Figs 1–3 and Table 2), the weightings assigned to included factors for which data was available were proportionally adjusted to sum to 100. High opportunity marine ecoregions based on the restorative aquaculture opportunity index scenario that included a synthesis of all environmental factors only is presented in panels (C) and (D), socioeconomic factors only (E), and human health factors only (F) according to their assigned weights. (TIFF) [file pone.0222282.s002.tiff]

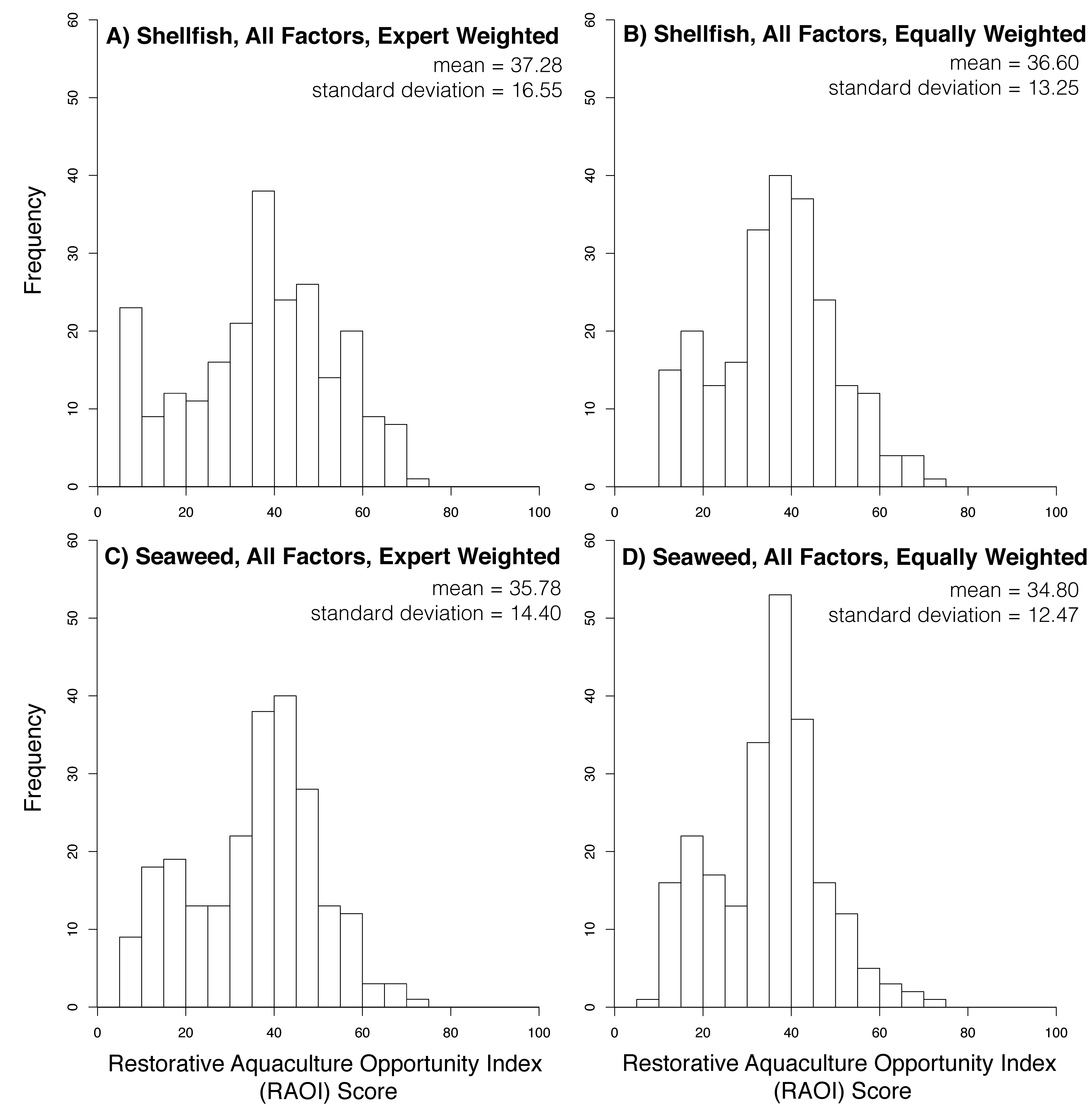

Supplement: S3 Fig — The mean and standard deviation of RAOI scores in each scenario are included. (TIF) [file pone.0222282.s003.tif]
